# Seismic Response of 3D Steel Buildings considering the Effect of PR Connections and Gravity Frames

**DOI:** 10.1155/2014/346156

**Published:** 2014-06-03

**Authors:** Alfredo Reyes-Salazar, Edén Bojórquez, Achintya Haldar, Arturo López-Barraza, J. Luz Rivera-Salas

**Affiliations:** ^1^Facultad de Ingeniería, Universidad Autónoma de Sinaloa, Ciudad Universitaria, Culiacán, 80040 Sinaloa, MEX, Mexico; ^2^Department of Civil Engineering and Engineering Mechanics, University of Arizona, Tucson, AZ 85721, USA

## Abstract

The nonlinear seismic responses of 3D steel buildings with perimeter moment resisting frames (PMRF) and interior gravity frames (IGF) are studied explicitly considering the contribution of the IGF. The effect on the structural response of the stiffness of the beam-to-column connections of the IGF, which is usually neglected, is also studied. It is commonly believed that the flexibility of shear connections is negligible and that 2D models can be used to properly represent 3D real structures. The results of the study indicate, however, that the moments developed on columns of IGF can be considerable and that modeling buildings as plane frames may result in very conservative designs. The contribution of IGF to the lateral structural resistance may be significant. The contribution increases when their connections are assumed to be partially restrained (PR). The incremented participation of IGF when the stiffness of their connections is considered helps to counteract the no conservative effect that results in practice when lateral seismic loads are not considered in IGF while designing steel buildings with PMRF. Thus, if the structural system under consideration is used, the three-dimensional model should be used in seismic analysis and the IGF and the stiffness of their connections should be considered as part of the lateral resistance system.

## 1. Introduction and Objectives

Different structural configurations, structural systems, and materials are generally used to improve structural behavior during seismic excitations. For steel buildings, the use of moment resisting frames (MRF) has been popular because they provide maximum flexibility for space utilization and because of their high ductility capacity. The seismic behavior of this structural system has been a research topic of interest in the profession during the last few decades. Foutch and Yun [1] investigated the accuracy of simple nonlinear as well as more detailed modeling methods used in the design of MRF. In another study, Lee and Foutch [2] studied the seismic behavior of 3-, 9-, and 20-story MRF designed for different reductions (R) factors. Krishnan et al. [3] determined the damage produced by hypothetical earthquakes on two 18-story MRF, one existing and one improved according to the 1997 Uniform Building Code [4], located in southern California, USA. Liao et al. [5] developed a three-dimensional finite-element model to examine the effects of biaxial motion and torsion on the nonlinear response of MRF. Effects of gravity frames, panel zones, and inelastic column deformation were considered. Kazantzi et al. [6] proposed a methodology for the probabilistic assessment of low-rise steel buildings and applied it to a welded MRF, emphasizing the modeling of connections. More recently, Chang et al. [7], by using 6- and 20-level steel office buildings, studied the role of accidental torsion in seismic reliability assessment. Bojórquez et al. [8] found that moment resisting steel plane frames are very efficient in dissipating earthquake-induced energy and that the dissipated energy has an important effect on the structural response. Garcia et al. [9] proposed a displacement-based design methodology for steel frame-RC wall structures. Their structural performance was investigated through nonlinear time-history analyses by using seven spectrum-compatible accelerograms. Black [10] by using analytical and regression analysis methods proposed two stability coefficients that can be used to quantify the P-Δ effect during elastic and inelastic lateral displacements of regular steel MRF. Black [11] used regression analysis of data collected from nonlinear static and modal analyses of 22 steel MRF to propose empirical equations to estimate key inelastic parameters, as frame's yield displacement and strength. Sejal et al. [12] compared the seismic response of a steel MRF designed by the performance-based plastic design method with that of conventional elastic design method based on the seismic evaluation done by both nonlinear static and nonlinear dynamic analyses under different ground motions.

The characteristics of the basic structural system of steel buildings with MRF have significantly changed over the years in some developed countries like the USA. From the mid-60s to the mid-70s, most connections in steel buildings were assumed to be fully restrained (FR). In the recent past, the use of FR connections was reduced considerably because they were expensive and to eliminate weak-axis connections [13]. FR connections are used only on two frame lines in each direction, usually at the perimeter (PMRF). As a result of this structural arrangement, the redundancy of the building is significantly reduced. An important issue that deserves our attention is that PMRF are usually designed as plane frames to resist the total lateral seismic loading, ignoring the presence of interior gravity frames (IGF), which are designed to resist only the gravity loads. Due to the action of the rigid floor diaphragm, the IGF, however, will undergo the same lateral deformation as the PMRF, developing bending moments and shear forces in columns. Therefore, the contribution of IGF to the lateral resistance of the building could be significant, particularly for buildings with relatively few FR connections. Moreover, modeling the buildings as plane frames may not represent the actual behavior of the structure since the participation of some elements is not considered and the contribution of some vibration modes is ignored.

Another simplification made in the design of steel buildings with PMRF and IGF is related to the stiffness of the beam-to-column connection. Conventional analysis and design of steel frames are based on the assumption that beam-to-column connections are either FR or perfectly pinned (PP). In the analysis and design of the above-mentioned structural system, the beam-to-column connections of the PMRF are assumed to be FR while those of IGF are assumed to be PP. Despite these classifications, almost all steel connections used in real steel buildings are essentially partially restrained (PR) with different rigidities. It has been established in the profession, both theoretically and experimentally, that these connections exhibit semirigid nonlinear behavior even if the applied loads are very small [14]. Modeling a connection to be either FR or PP type is simply an assumption made to simplify calculations and is a major weakness in current analytical procedures. These simplifications may result in erroneous values for resultant stresses because in reality FR connections possess some flexibility and PP connections possess some rigidity. The contribution of these connections to the structural strength and stiffness can be very important if the composite action of the concrete slab is considered [15, 16]. Even though it is not an objective of this paper to compare the seismic responses of steel buildings with PR and FR connections, it is also important to mention that in some studies [17–21] it was shown that the maximum values of base shear and interstory displacements of plane steel frames under earthquake ground motions were reduced when PR connections were used. The reason for this is that the frames with PR connections dissipate more energy and attract less inertial forces than frames with FR connections. The efficiency of PR connections has been also studied in other investigations. MacRae et al. [22] showed, for steel concentrically braced frames (generally designed to resist lateral force by means of truss action), that columns are generally continuous and that they possess some flexural stiffness and strength which significantly decrease the possibility of large drift concentrations. Kishi et al. [23] investigated the reduction in costs of tall buildings with mixed FR and PR connections. Kishi at al. [24] proposed a useful design aid for determining the values of the connection parameters with the help of a set of monographs which allows the engineer to rapidly determine the moment-rotation curve for a given connection. In spite of the important contributions of the above-mentioned studies, structural subassemblies or plane frames were considered.

The state-of-the-art report “seismic performance of steel moment frames subjected to earthquake ground motion shaking” [13] under the leadership of Professor Krawinker represents a major step in the advance of the understanding of the seismic behavior of steel buildings. However, as stated in the report itself, the study on the effect of interior gravity columns and their shear connections and columns of the orthogonal moments frames on the seismic response was limited since this effect was approximately considered by using a 2D model and a single column (“flag pol”). In the paper under evaluation, all interior gravity columns and all their connections are explicitly considered in a 3D model. The loading and unloading processes at the PR connections and, consequently, the dissipated energy are explicitly considered too.

The above discussions clearly indicate that there are several issues that need our attention regarding the structural idealization of steel buildings with PMRF and IGF. The particular issues addressed in this study are (a) to estimate the relative importance of the bending moments developed in gravity frames, (b) to evaluate the contribution of the IGF as well as that of the “usually neglected” stiffness of their connections to the lateral structural resistance, (c) to evaluate the accuracy of modeling the three-dimensional (3D) steel buildings as two-dimensional (2D) frames for seismic analysis, and (d) to compare the seismic responses of 3D buildings with PP and PR connections. Some steel structures that satisfy all the current seismic requirements proposed in the SAC steel project [13] and several recorded strong motion earthquakes are used for this purpose. An assumed-stress-based finite element algorithm, developed and implemented by the authors and other research team members in a computer program, is used to estimate the responses. The procedure can consider material and geometric nonlinearities and that produced by partially restrained (PR) connections.

## 2. Mathematical Formulation

### 2.1. The Assumed-Stress-Based Finite Element Algorithm

Estimation of linear and nonlinear responses in time domain for 3D realistic structures excited simultaneously by all three components of an earthquake is essential to meet the objectives of the study. As discussed earlier and commented on in more detail later, several model steel buildings suggested in the SAC steel project [13] will be used for numerical evaluations to study the above-mentioned objectives. To estimate the responses of three-dimensional moment resisting steel frames in the presence of all major sources of nonlinearities, the assumed-stress-based finite element algorithm is found to be very accurate and efficient [25, 26]. In this approach, an explicit form of the tangent stiffness matrix is derived without any numerical integration. Fewer elements can be used in describing a large deformation configuration without sacrificing any accuracy, and the material nonlinearity and geometric nonlinearities and that introduced by PR connections can be incorporated without losing its basic simplicity. The authors and their colleagues developed the theoretical concept to estimate nonlinear seismic responses considering geometric and material nonlinearities and several major sources of energy dissipation in steel frame structures. They wrote a computer program to implement the concept. The procedure and the algorithm were verified using available theoretical and experimental results [14, 15]. Only the basic concepts of the procedure are presented here.

The linear iterative strategy used to solve the nonlinear dynamic equation of motion can be expressed as

(1) m(t+Δt)U¨(k)+C(t+Δt)tU˙(k)+K(t+Δt)tΔU(k)  =  (t+Δt)F(k)−(t+Δt)R(k−1)−mU¨g(k),

where **m**, **C**, and ^*t*^ **K** are the mass, damping, and the tangent stiffness matrices, respectively, $\ddot{U}$ and $\dot{U}$ are the acceleration and velocity vectors, respectively, Δ**U** is the incremental displacement vector, **F** is the external load vector, **R** is the internal force vector, and $\ddot{U_{g}}$ is the ground acceleration vector. Superscripts (*t* + Δ*t*) and (*k*) indicate the time and the iteration number, respectively. Rayleigh-type damping is commonly used for nonlinear analysis in the profession since it is a function of the mass and stiffness matrices representing the current state of a structure. Rayleigh-type damping is considered in this study.

Explicit expressions for the tangent stiffness matrix and the internal force vector are developed for each beam-column element for the *k*th iteration at time *t*. The nonlinear elastic tangent stiffness matrix for a beam-column element, **K** ^*e*^, can be represented as

(2) Ke=AσdoTAσσ−1Aσdo+Addo,

where **A**_*σσ*_^−1^ is the elastic property matrix, **A**_*σdo*_ is the transformation matrix, and **A**_*ddo*_ is the geometric stiffness matrix. Similarly, the internal force vector of an element, **R** ^*e*^, can be expressed as

(3) Re=−AσdoTAσσ−1Rσ+Rdo,

where **R**_*do*_ is the homogeneous part of the internal force vector and **R**_*σ*_ is the deformation difference vector.

The nonlinear structural behavior discussed above also needs to be modified to consider material nonlinearity. In this study, the material is considered to be linear elastic except at plastic hinges. Concentrated plasticity behavior is assumed at plastic hinge locations. For mathematical modeling, plastic hinges are assumed to occur at locations where the combined action of axial force, torsion, and bending moments satisfies a prescribed yield function. This is discussed in detail elsewhere [25]. The yield function for three-dimensional beam-column elements and W-shape sections used in this study has the following form:

(4) (PPn)2+(MXMnX)2+(MYMnY)2+(MZMnZ)2−1=0,

where *P* is the axial force, *M*_*X*_ and *M*_*Y*_ are the bending moments with respect to the major and minor axes, respectively, *M*_*z*_ is the torsional moment,*P*_*n*_ is the axial strength, *M*_*nX*_ and *M*_*nY*_ are the flexural strength with respect to the major and minor axes, respectively, and *M*_*nZ*_ is the torsional strength. The presence of plastic hinges in the structure will produce additional axial deformation and relative rotation in a particular element. Thus, the tangent stiffness matrix needs to be modified if plastic hinges form. The elastoplastic tangent stiffness matrix **K**_*P*_ and the elastoplastic internal force vector **R**_*P*_ can be obtained by modifying the corresponding elastic matrixes as [27, 28]

(5) KPe=Ke−AσdoTAσσ−1VPCPTAσdo,RPe  =  AσdoT(Aσσ−1VPCPT−Aσσ−1)R^σ+Rdo.

In (5), **V**_*P*_, **C**_*P*_, and ${\hat{R}}_{\sigma }$ can be shown to be

(6) VP=[−∂f∂N,−∂f∂M(1−xl),−∂f∂M(xl)]T,

(7) CPT  =  (VPTAσσ−1)−1VP−1Aσσ−1,

(8) R^σ  =  Rσ+{HPθP∗(1−Xl)  θP∗(Xl)},

where *H*_*P*_ and *θ*_P_* in (8) are the additional axial elongation and additional relative rotation at plastic hinges.

Depending on the level of earthquake excitation, all the elements in a typical structure may remain elastic or some of the elements may remain elastic and the rest may yield. As stated earlier, the structural stiffness matrix can be explicitly obtained by considering individual elements and the corresponding element stiffness matrixes, depending on the particular state they are in. Equations (2) and (3) can be used if a particular element is in the elastic state. Equations (5) should be used if the element has yielded. The procedure is discussed in more detail elsewhere [25, 27, 29].

### 2.2. Rayleigh Damping

Since actual earthquake time histories are used in this study, the inertia and applied forces are available. However, the damping is an important parameter which needs further discussion at this stage. In a realistic seismic analysis of steel frames, the amount of damping energy that will be generated will depend on the nonyielding and yielding states of the material and on the hysteretic behavior if the material yields. For mathematical simplicity, the effect of nonyielding energy dissipation is usually represented by equivalent viscous damping varying between 0.1% and 7% of the critical damping. The damping is often increased in linear analysis to approximate energy losses due to anticipated inelastic behavior [30]. In a rigorous seismic analysis this practice is not appropriate, since the energy losses due to inelastic behavior would be counted twice. Based on an extensive literature review, it is observed that the following Rayleigh-type damping is very commonly used in the profession:

(9) $$\begin{array}{c} {}^{t}C=\alpha m+\gamma^{t}K, \end{array}$$

where *α* and *γ* are the proportional constants. The use of both the tangent stiffness and the mass matrices is a very rational approach to estimate the energy dissipated by viscous damping in a nonlinear seismic analysis. The constants *α* and *γ* can be determined from specified damping ratios *ξ*_*i*_ and *ξ*_*j*_ for the *i*th and *j*th modes, respectively. Then the following algebraic equation system is solved for *α* and *γ* [31]:

(10) [1ωiωi1ωjωj]{αγ}=2{ξiξj}.

### 2.3. The Newmark *β* Method

The step-by-step direct integration numerical analysis procedure using the Newmark *β* method is used to solve (1). The displacements and velocity vectors within each time step Δ*t* are assumed as follows [32]:

(11) (t+Δt)D˙(k)=D˙t+((1−η)D¨t+ηD¨(k)(t+Δt))Δt,

(12) (t+Δt)D(k)=Dt+D˙tΔt +((12−β)D¨t+β(t+Δt)D¨(k))Δt2,

where *η* and *β* are the parameters which need to be determined to obtain the integration accuracy and stability. In this study *η* = 1/2 and *β* = 1/4 are used. For these values, the acceleration vector is constant within each interval Δ*t*, and the method is considered to be unconditionally stable.

It is assumed that the displacement and dynamic force vectors of the *k*th iteration at time *t* + Δ*t* can be expressed in incremental form as

(13) D(k)(t+Δt)=D(k−1)(t+Δt)+ΔD(k)(t+Δt),F(k)(t+Δt)=F(k−1)(t+Δt)+ΔF(k)(t+Δt).

Substituting (9), (11), and (12) into (1), manipulating and assembling some terms together, and using (13), the following governing equation results:

(14) KDtΔD(k)(t+Δt) =FD(k−1)(t+Δt)+ΔFD(k)(t+Δt)−R(k−1)(t+Δt),

where ^*t*^ **K**_**D**_ = the modified tangent stiffness matrix, ^(*t* + Δ*t*)^ **F**_**D**_^(*k*−1)^ = the modified external force vector, and ^(*t* + Δ*t*)^Δ**F**_**D**_^(*k*)^ = the incremental force vector.

^*t*^ **K**_**D**_, ^(*t* + Δ*t*)^ **F**_**D**_^(*k*−1)^, and ^(*t* + Δ*t*)^Δ**F**_**D**_^(*k*)^ have the following form:

(15) KDt=f1M+f2Kt,

(16) FD(k−1)(t+Δt)=F(k−1)(t+Δt)+p(k−1)(t+Δt) −MD¨(k−1)(t+Δt),

(17) ΔFD(k)(t+Δt)=ΔF(k−1)(t+Δt)−MΔD¨g(k)(t+Δt).

The term ^(*t* + Δ*t*)^ **p** ^(*k*−1)^ in (16) is the modified force vector contributed by the displacement, velocity, and acceleration vectors at time *t* and the displacement vector at time *t* + Δ*t* and can be written as

(18) p(k−1)(t+Δt)=M(f1Dt+f3D˙t+f4D¨t−f1D(k−1)(t+Δt))+Kt(f5Dt+  f6D˙t+  f7D¨t     −f5D(k−1)(t+Δt)).

The coefficients **f**_*i*_ can be evaluated in terms of *η*, *β*, *α*, *γ*, and Δ*t* as

(19) f1=1βΔt2+ηαβΔt,f2=ηγβΔt+1,f3=1βΔt+ηαβ−α,f4=(12β−1)+ηα(12β−1η)Δt,f5=ηαβΔt,f6=ηγβ−γ,f7=(ηγ2β−γ)Δt.

Equation (14) needs to be solved. A computer program has been developed for this purpose. The program was extensively verified using information available in the literature. The structural response behavior and the members' forces in terms of axial load, shear force, and bending moment can be estimated using the computer program.

### 2.4. The Richard Model

Connections are structural elements that transmit resultant stresses between beams and columns. For the case of PR connections, their rigidity is generally represented by the bending moment acting on them and the corresponding relative rotation. Many mathematical forms to define the bending moment-relative rotation relationship (referred to as *M*-*θ* curve) for PR connections are available in the literature. They include the piecewise linear, the polynomial, the exponential, the B-spline, and the Richard model [29, 33]. The Richard model is a four-parameter model which was developed using actual worldwide test data and is adopted in this study.

When a connection is defined in terms of member sizes, bolts, and/or welds, a commercially available computer program, known as PRCONN, is available to generate the appropriate *M*-*θ* curve using the Richard model [33]. This program is used in this study to develop the required *M*-*θ* curve. According to the Richard model, the *M*-*θ* curve is given by

**Figure 1:**
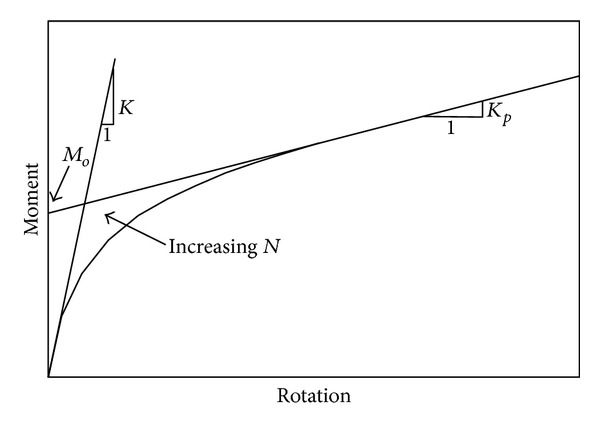
Parameters of Richard's model.

(20) M=(K−KP)θ(1+|(K−KP)θ/M0|N)1/N+Kpθ,

where *K* is the initial or elastic stiffness, *K*_*P*_ is the plastic stiffness, *M*_0_ is the reference moment, and *N* is the curve shape parameter. The loading process and the physical definition of these parameters are shown in Figure 1. The term “increasing *N*” in this figure means that the *M*-*θ* curve tends to be bilinear as *N* increases.

Equation (20) represents the *M*-*θ* curve when the load is increasing monotonically. When a structure is excited by dynamic or seismic loading, some of the connections are expected to be loading and others are expected to be unloading and reloading. Experimental and theoretical studies related to the unloading and reloading behaviors of the *M*-*θ* curve are rare. This subject has been addressed in the literature [34, 35]. For the present study, the unloading and reloading behaviors of the *M*-*θ* curves are essential. As in other studies [14, 15, 29, 36, 37], in the present study, the monotonic loading behavior is represented by the Richard curve and the Masing rule is used to theoretically develop the unloading and reloading sections of the *M*-*θ* curves. Using the Masing rule and the Richard model represented by (20), the mathematical representation for the unloading and reloading behaviors of a connection can be expressed as

(21) M=Ma−(K−KP)(θa−θ)(1+|(K−KP)(θa−θ)/2M0|N)1/N−KP(θa−θ).

**Figure 2:**
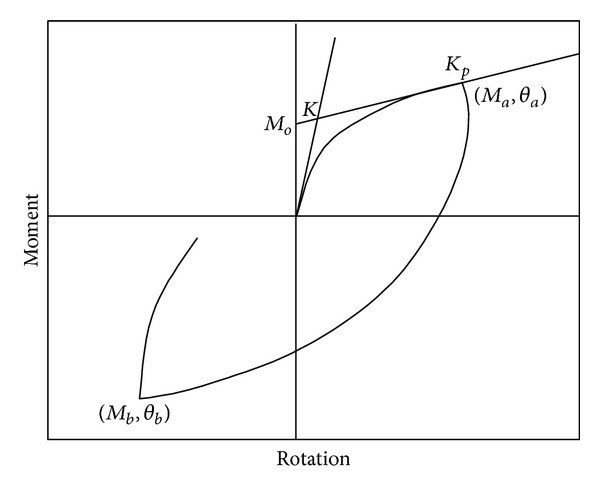
Loading, unloading, and reloading at PR connections.

The loading, unloading, and the reloading at PR connections are illustrated in Figure 2. If (*M*_*b*_, *θ*_*b*_) is the next reversal point, as shown in the figure, the reloading relation between *M* and *θ* can be obtained by simply replacing (*M*_*a*_, *θ*_*a*_) with (*M*_*b*_, *θ*_*b*_) in (21). Thus, (20) is used if the connection is loading; if it is unloading or reloading, (21) should be used instead.

### 2.5. Structural Models

**Figure 3:**
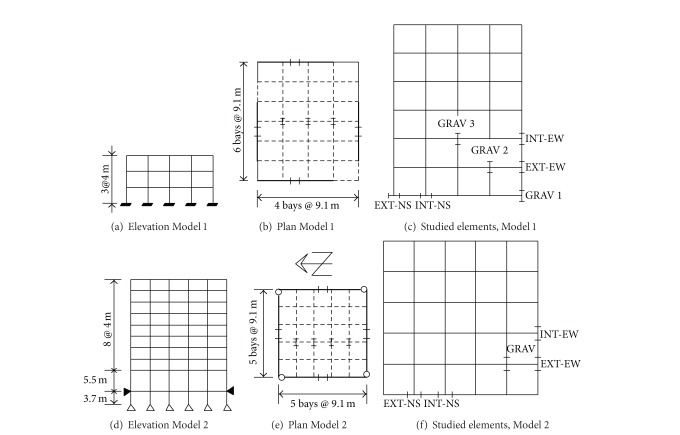
Elevation, plan, and element location for Models 1 and 2.

As part of the SAC steel project [13], three consulting firms were commissioned to design 3-, 9-, and 20-story buildings. They were designed to satisfy the code requirements of Los Angeles [4], Seattle [4], and Boston [38]. The 3- and 9-story buildings located in the Los Angeles area with the pre-Northridge designs are considered to address all the issues raised earlier. They will be denoted hereafter as Models 1 and 2, respectively. The fundamental periods of the buildings are 1.03 and 2.34 sec, respectively. The elevation and plan of both models showing the location of MRF (continuous lines) and the orientation of elements specifically considered in the study are shown in Figure 3. The sizes of the beams and columns of the models are given in Table 1. The columns of the PMRF of Model 1 are considered to be fixed at the base while those of Model 2 are assumed to be pinned. In both models the columns of gravity frames are pinned at the base. In all these frames, the columns are assumed to be made of grade-50 steel and the girders are of A36 steel. All the columns in the PMRF bend about the strong axis. The strong axis of the gravity columns is oriented in the N-S direction. The designs of the PMRF in the two orthogonal directions were practically the same. The slab was modeled by near-rigid struts, as considered in the FEMA study. Additional information for the models can be obtained from the SAC steel project report [13].

The frames are modeled as complex multi-degree of freedoms (MDOF) systems in this study. Each column is represented by one element and each girder of the PMRF is represented by two elements, having a node at the midspan. Each node is considered to have six degrees of freedom to capture their three-dimensional behavior. The total number of degrees of freedom is 846 and 3408, for Models 1 and 2, respectively. The models are excited by twenty recorded earthquake time histories measured at different locations. The details of the time histories are given in Table 2. They were obtained from the data sets of the national strong motion program (NSMP) of the United States Geological Surveys (USGS). Additional information regarding the earthquakes can be obtained from them. The damping in the models is considered to be 5% of the critical; the same damping is used in the codified approaches.

## 3. Results and Discussion

### 3.1. Relative Magnitude of the ****p****-**δ** Effect in Gravity Columns

In order to estimate the relative importance of the moments (*M*_**δ**_) produced on gravity columns by seismic lateral loading and the axial loads (**p**-**δ** effect), the *M* parameter, defined as the ratio of this moment (*M*_**δ**_) and a moment defined as a reference moment (*M*_*G*_), is estimated. The reference moment is that used in concrete columns defined as the “minimum design moment” and it is adopted here for steel columns only for comparison purposes. *M*_**δ**_ is calculated from time-history analysis of the three-dimensional steel buildings with PP connections and *M*_*G*_ is assumed to be given by

(22) MG  =  PU(1.5+0.03h),

where *P*_*U*_ represents the gravity axial load and *h* the depth of the columns.

In the seismic analysis, the different earthquake acceleration records are first normalized with respect to the pseudoacceleration evaluated at the fundamental structural period (*S*_*a*_(*T*_1_)); in other words, for a given model, the earthquakes are scaled up or down in such a way that the ordinate values of their elastic pseudoacceleration response spectra, evaluated at the fundamental period (*T*_1_) of the model, are the same for all the records.

The frames did not develop any plastic hinge when excited by any of the 20 recorded earthquakes. To study the effect of inelastic behavior, the actual time histories were scaled up so that yielding was produced in the models. Based on the past experience and for the uniformity of comparison, all the actual time histories were scaled up to develop a maximum average interstory drift of about 2% by the trial and error procedure, instead of tracking the total number of plastic hinges developed. It was observed that about 8 to 16 plastic hinges were formed in the models when they develop the desired drift.

Values of the *M* parameter are estimated for corner and interior gravity columns, for elastic and inelastic behaviors, and for the E-W and N-S excitations. Only the results in terms of the basic statistics (mean *μ*, standard deviation *σ*, and coefficient of variation *δ*) for Model 1 and three gravity columns (see Figure 3(c)) are reported; they are given in Table 3. The most important observation that can be made is that the values of *M* are close and even larger than unity in most of the cases indicating that the magnitude of the moments developed in gravity columns can be considerable and, consequently, should not be neglected. The values of *M* for Model 2 are similar to those of Model 1.

### 3.2. Contribution of IGF

The contribution of IGF to the lateral resistance, in terms of interstory shears for the models with PP connections, is first discussed. The shear ratio *V*_1_, defined as *V*_*G*_/*V*_*T*_, is introduced for this purpose. For a given direction and story, *V*_*G*_ will represent the lateral shear resisted by all the IGF and *V*_*T*_ will represent the total lateral shear.

**Figure 4:**
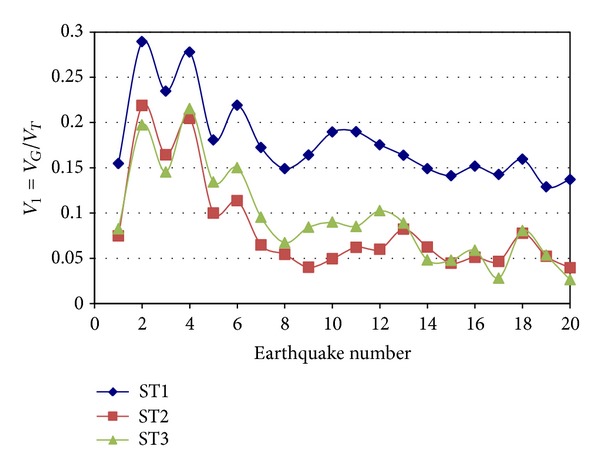
Ratio of IGF shear to total shear, Model 1, E-W direction.

**Figure 5:**
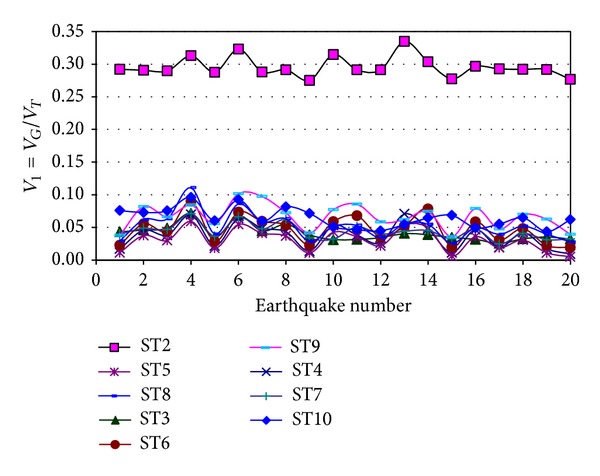
Ratio of IGF shear to total shear, Model 2, E-W direction.

Typical results of the *V*_1_ parameter are shown in Figures 4 and 5 for Models 1 and 2, respectively, for the E-W direction and elastic behavior. The symbol *ST* is used to represent the word “story.” It is observed that the *V*_1_ values significantly vary from one model to another and from one story to another without showing any trend. Values of up to 29% are obtained for Story 1 of Model 1. Similar plots to those of Figures 4 and 5 were also developed for the N-S direction but are not shown. The major observations made before are also valid for this direction. The results for inelastic behavior were also estimated; the only additional observation that can be made is that the *V*_1_ values are larger for inelastic behavior than for elastic behavior for some particular cases. For most of the cases however, they are quite similar since yielding was not too significant.

The statistics of *V*_1_ are summarized in Table 4. As observed from individual values of *V*_1_, the statistics also indicate that the IGF can significantly contribute to the lateral resistance. It is also observed from the table that the uncertainty associated with the estimation, in terms of *δ*, is relatively high. Based on the above results, it is concluded that the contribution of the IGF to the lateral resistance should not be overlooked in the design of the structural systems under consideration.

**Figure 6:**
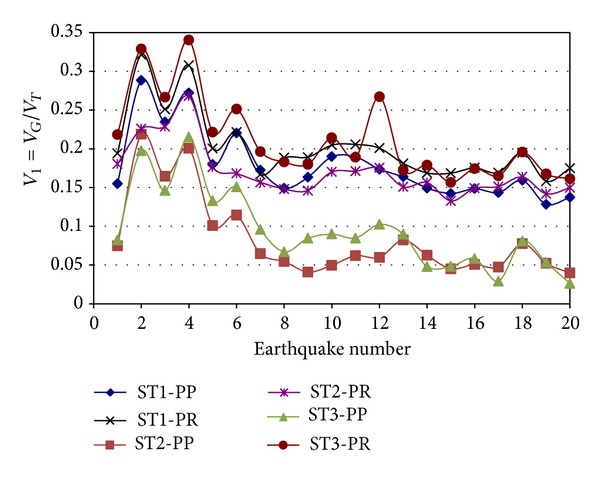
Ratio of IGF shear to total shear, PP and PR connections, Model 1, E-W direction.

The effect of the connection stiffness of the IGF on the *V*_1_ parameter is now estimated. The results for Model 1 and the E-W direction are presented in Figure 6 for both PP and PR connections. It is observed that the contribution of the IGF to the lateral resistance significantly increases when the stiffness of the connections is considered. The increment is particularly important for the upper stories. For example, for Story 3, *V*_1_ was smaller than 0.12 in most of the cases for the frames with PP connections. For PR connections however, this parameter takes values larger than 0.20 in most of the cases. Values close to 0.30 are observed for Story 3 for two cases. These results indicate that the contribution of IGF to the lateral resistance is not negligible and cannot be ignored in the analysis and design of members, particularly the columns, of the IGF. The major implication is that the members in the IGF may not be able to carry these unexpected load effects due to nonnegligible lateral load.

### 3.3. Accuracy of Using 2D Models

**Figure 7:**
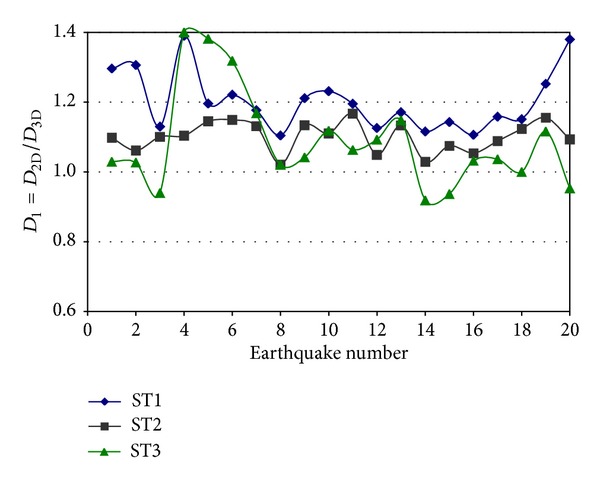
Ratio of displacements for 2D and 3D models, Model 1, E-W direction.

The seismic responses of the steel buildings modeled as 2D structures are compared with those of the more realistic 3D structural representations; PP connections are assumed in the IGF. The interstory displacements are considered first. The *D*_1_ parameter, defined as *D*_2D_/*D*_3D_, is introduced for this purpose. For a given story and direction, *D*_2D_ represents the maximum displacement of the story under consideration when the building is modeled as a plane frame while *D*_3D_ represents the same but for the building modeled as a 3D structure. The *D*_1_ ratio is estimated for both horizontal directions. Typical values of *D*_1_ are presented in Figure 7 for Model 1, the E-W direction, and elastic behavior. It is observed that the *D*_1_ values significantly vary from one model to another and from one story to another without showing any trend. In most of the cases they are larger than unity indicating that the interstory displacements are larger for the 2D model than for the 3D model. Values of up to 1.4 are observed in some cases. The *D*_1_ values are larger, in general, for the story at ground level. The differences between the responses of the 2D and 3D models point out that the dynamic characteristics of 2D and 3D structural representations are different and they just cannot be overlooked. It is well known that the response of three-dimensional buildings when subjected to strong motions depends on many factors, specifically on the spatial distribution of strength, stiffness, and mass, the frequency content of the excitation, and the energy dissipation characteristics (damping) in the linear and nonlinear responses. It is important to emphasize that a building modeled as a 3D frame is expected to have different natural frequencies than that modeled as a 2D frame and will respond differently when subjected to the same excitation.

**Figure 8:**
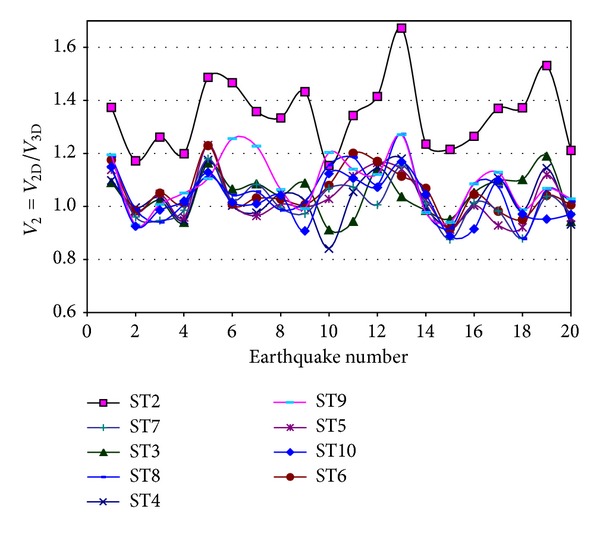
Ratio of shears for 2D and 3D models, Model 2, E-W direction.

A similar ratio (*V*_2_) to that of interstory displacements is also estimated for interstory shears. The results are presented in Figure 8 for Model 2, the E-W direction, and elastic behavior. As for the case of the *D*_1_ parameter, the values of *V*_2_ are, in general, larger than unity in most of the cases, varying from one model and one story to another without showing any trend.

Plots similar to those of elastic analysis (Figures 7 and 8) for *D*_1_ and *V*_2_ are also developed for inelastic behavior; only their statistics are given (Table 5). It is observed that on an average basis their values are quite similar for elastic and inelastic behaviors. Results also indicate that the uncertainty in the estimation, in terms of coefficients of variation, is not large.

Based on the earlier results, it is concluded that modeling the structural systems under consideration as plane frames may significantly overestimate the seismic response; in other words, it will produce conservative design.

### 3.4. Response of the 3D Models with PP and PR Connections

**Figure 9:**
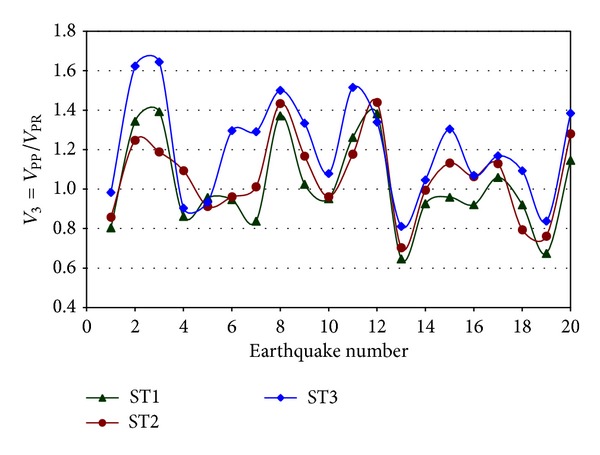
Ratio of shears for the model with PP and PR connections for an exterior frame of Model 1, E-W direction.

The effect of the stiffness of the beam-to-column connections of the IGF on the overall structural response, in terms of the ratio of the interstory shear of the buildings with PP connections to that of the building with PR connections, is discussed in this part of the paper. The *V*_3_ parameter, defined as *V*_PP_/*V*_PR_, is used for this purpose. Only results for Model 1 are presented. For a given story, *V*_PP_ will represent the shear on that story when PP connections are considered in the IGF. *V*_PR_ will represent the same, except that PR connections are used instead. Results for an exterior MRF oriented in the E-W direction are shown in Figure 9. It is observed that the *V*_3_ values are in most of the cases larger than unity indicating that the interstory shears of the exterior frame are larger for the model with PP connections when compared with those of the model with PR connections. The reason for this is that the contribution to the building lateral resistance of the IGF when PP connections are considered is relatively small since it is provided only by the exterior columns which are part of the transversal MRF located in the transversal (N-S) direction. Therefore, the lateral overall building resistance is mostly provided by the exterior frames. On the other hand, the contribution of the IGF to the lateral resistance is significantly increased when the PR connections are considered; therefore the contributionof the exterior frames (*V*_PR_) decreases.

**Figure 10:**
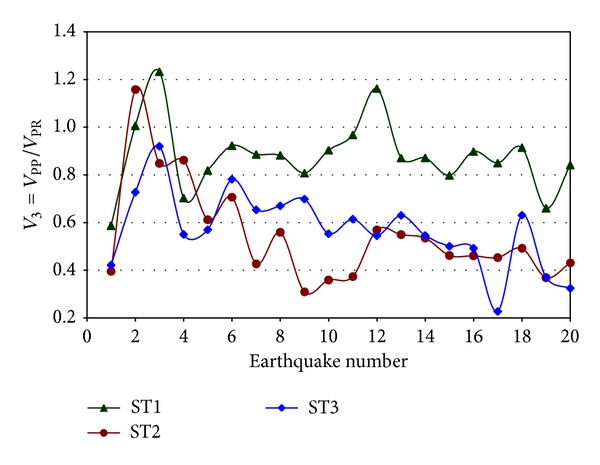
Ratio of shears for buildings with PP and PR connections for an interior frame of Model 1, E-W direction.

The values of *V*_3_ for an interior frame (IGF) oriented in the E-W direction are shown in Figure 10. Results indicate that they are smaller than unity practically in all the cases. As discussed earlier, the contribution to the lateral resistance (*V*_PP_) of the interior frames of the model with PP connections is smaller than that (*V*_PR_) of the model with PR connections. Thus, the incremented participation of IGF when the stiffness of the connections is considered helps counteract the no conservative effect that results in practice when lateral seismic loads are not considered in IGF while designing steel buildings with PMRF.

It is commonly believed by structural engineers, at least in México, that the flexibility of shear connections (like those used in IGF of the structural systems under consideration) is negligible and that 2D models can be used to properly represent 3D real structures. The results presented in Sections 3.1 and 3.3 of this paper indicate, however, that the moments developed in columns of IGF can be considerable and that modeling buildings as plane frames may result in very conservative designs. The differences between the seismic responses of the 3D buildings with PP connections and the 3D buildings with PR connections or the 2D models point out that the dynamic characteristics of these structural representations are different and they just cannot be overlooked.

## 4. Conclusions

The nonlinear seismic responses of steel buildings with perimeter moment resisting frames (PMRF) and interior gravity frames (IGF) are studied, modelling them as complex three-dimensional (3D) structures and explicitly considering the contribution of the IGF and their connections. Two building models, used in the SAC steel project, are considered in the study. The models are excited by several recorded strong motion earthquakes and the linear and nonlinear responses for different structural idealizations are calculated. It is commonly believed by structural engineers that the flexibility of shear connections is negligible and that bidimensional (2D) models can be used to properly represent 3D real structures. The results of this paper indicate, however, that the moments developed in columns of IGF can be considerable and that modeling buildings as plane frames may result in very conservative designs. The results of the study also indicate that the contribution of IGF to the lateral structural resistance may be significant. For the case of perfectly pinned (PP) connections, this contribution is larger for lower stories. The contribution increases when the connections of the IGF are assumed to be partially restrained (PR), particularly for upper stories. The incremented participation of IGF when the stiffness of the connections is considered helps counteract the no conservative effect that results in practice when lateral seismic loads are not considered in IGF while designing steel buildings with PMRF and IGF. The differences between the seismic responses of the 3D buildings with PP connections and the 3D buildings with PR connections or the 2D model point out that the dynamic characteristics of these structural representations are different and they just cannot be overlooked. It is well known that the response of a building when subjected to strong motions depends on many factors, specifically on the spatial distribution of strength, stiffness, and mass, the frequency content of the excitation, and the energy dissipation characteristics. It is important to emphasize that a building modeled as a 3D frame is expected to have different natural frequencies than a building modeled as a 2D frame and will respond differently when subjected to the same excitation. Thus, if the structural system under consideration is used, the three-dimensional model should be used in seismic analysis and the IGF and the stiffness of their connections should be considered as part of the lateral resistance system.

## Figures and Tables

**Table 1:** Beam and column sections for Models 1 and 2.

Model	Moment resisting frames				Gravity frames		
	Story	Columns		Girders	Columns		Beams
		Exterior	Interior		Below penthouse	Others	
3-level	1/2	*W*14 × 257	W14 × 311	W33 × 118	W14 × 82	W14 × 68	W18 × 35
	2/3	W14 × 257	W14 × 312	W30 × 116	W14 × 82	W14 × 68	W18 × 35
	3/Roof	W14 × 257	W14 × 313	W24 × 68	W14 × 82	W14 × 68	W16 × 26

10-level	−1/1	W14 × 370	W14 × 500	W36 × 160	W14 × 211	W14 × 193	W18 × 44
	1/2	W14 × 370	W14 × 500	W36 × 160	W14 × 211	W14 × 193	W18 × 35
	2/3	W14 × 370	W14 × 500, W14 × 455	W36 × 160	W14 × 211, W14 × 159	W14 × 193, W14 × 145	W18 × 35
	3/4	W14 × 370	W14 × 455	W36 × 135	W14 × 159	W14 × 145	W18 × 35
	4/5	W14 × 370, W14 × 283	W14 × 455, W14 × 370	W36 × 135	W14 × 159, W14 × 120	W14 × 145, W14 × 109	W18 × 35
	5/6	W14 × 283	W14 × 370	W36 × 135	W14 × 120	W14 × 109	W18 × 35
	6/7	W14 × 283, W14 × 257	W14 × 370, W14 × 283	W36 × 135	W14 × 120, W14 × 90	W14 × 109, W14 × 82	W18 × 35
	7/8	W14 × 257	W14 × 283	W30 × 99	W14 × 90	W14 × 82	W18 × 35
	8/9	W14 × 257, W14 × 233	W14 × 283, W14 × 257	W27 × 84	W14 × 90, W14 × 61	W14 × 82, W14 × 48	W18 × 35
	9/Roof	W14 × 233	W14 × 257	W24 × 68	*W*14 × 61	*W*14 × 48	*W*16 × 26

**Table 2:** Earthquake records.

Number	Place	Year	Station	*T* (seg.)	ED (km)	*M*	PGA (mm/seg^2^)
1	1317 Mich., México	1985	Paraíso	0.11	300	8.1	800
2	1634 Mammoth Lakes, USA	1980	Mammoth H.S. Gym	0.12	19	6.5	2000
3	1634 Mammoth Lakes, USA	1980	Convict Creek	0.19	18	6.5	3000
4	1317 Mich., México	1985	Infiernillo N-120	0.21	67	8.1	3000
5	1317 Mich., México	1985	La Unión	0.32	121	8.1	1656
6	1733 El Salvador	2001	Relaciones Ext.	0.34	96	7.8	2500
7	1733 El Salvador	2001	Relaciones Ext.	0.41	95	7.8	1500
8	1634 Mammoth Lakes	1980	Long Valley Dam	0.42	13	6.5	2000
9	2212 Denali Fault, AK	2000	K2-02	0.45	281	7.9	115
10	0836 Yountville, CA	2000	Redwood City	0.46	95	5.2	90
11	0408 Dillon, MT	2005	MT: Kalispell	0.51	338	5.6	51
12	1317 Mich., Mexico	1985	Villita	0.53	80	8.1	1225
13	1232 Northridge	1994	Hall Valley	0.54	25	6.4	2500
14	2115 Morgan Hill	1984	Hall Valley	0.61	14	6.2	2000
15	2212 Denali Fault, AK	2002	K2-04	0.62	290	7.9	133
16	0836 Yountville, CA	2000	Deauville F.S. Ca	0.63	73	5.2	144
17	0836 Yountville, CA	2000	Pleasant Hill F.S. 1	0.71	92	5.2	74
18	0836 Yountville, CA	2000	Pleasant Hill F.S. 2	0.75	58	5.2	201
19	2212 Denali Fault, AK	2002	Valdez City Hall	0.85	272	7.9	260
20	1715 Parkfield	2004	CA: Hollister City Hall	1.01	147	6	145

**Table 3:** Statistics of the ratio (*M*) of moments on gravity columns and the reference moment, Model 1.

Behavior	Story	GRAV 1						GRAV 2						GRAV 3					
		N-S			E-W			N-S			E-W			N-S			E-W		
		*μ*	σ	*δ*	*μ*	σ	*δ*	*μ*	σ	*δ*	*μ*	σ	*δ*	*μ*	σ	*δ*	*μ*	σ	*δ*
Elastic	1	0.71	0.12	0.17	0.84	0.27	0.32	0.65	0.11	0.17	0.76	0.27	0.36	0.71	0.12	0.17	0.74	0.27	0.36
	2	1.07	0.20	0.19	1.24	0.40	0.32	1.08	0.22	0.20	1.17	0.41	0.35	0.97	0.22	0.23	1.04	0.40	0.38
	3	1.09	0.20	0.18	1.34	0.39	0.29	1.15	0.24	0.21	1.13	0.42	0.37	1.17	0.25	0.21	1.14	0.39	0.34

Inelastic	1	0.75	0.12	0.16	0.87	0.30	0.34	0.94	0.11	0.12	0.87	0.29	0.33	0.78	0.12	0.15	0.97	0.30	0.31
	2	1.17	0.20	0.17	1.39	0.35	0.25	1.38	0.22	0.16	1.28	0.36	0.28	1.38	0.22	0.16	1.39	0.35	0.25
	3	1.19	0.20	0.17	1.49	0.35	0.23	1.28	0.24	0.19	1.38	0.39	0.28	1.48	0.25	0.17	1.49	0.35	0.23

**Table 4:** Statistics of the ratio (*V*_1_) of IGF shear to total shear.

Model	Story	N-S direction			E-W direction		
		*μ*	σ	*δ*	*μ*	σ	*δ*
1	1	0.22	0.07	0.31	0.18	0.02	0.13
	2	0.10	0.07	0.70	0.12	0.04	0.41
	3	0.11	0.07	0.60	0.11	0.04	0.45

2	2	0.21	0.01	0.06	0.30	0.02	0.05
	3	0.04	0.01	0.18	0.04	0.01	0.30
	4	0.03	0.02	0.65	0.04	0.02	0.52
	5	0.02	0.01	0.67	0.03	0.02	0.55
	6	0.03	0.01	0.40	0.05	0.02	0.45
	7	0.03	0.01	0.22	0.04	0.01	0.28
	8	0.03	0.01	0.41	0.05	0.02	0.41
	9	0.04	0.01	0.24	0.07	0.02	0.29
	10	0.04	0.01	0.33	0.06	0.02	0.23

**Table 5:** Statistics for the *D*_1_ and *V*_2_ parameters.

	Model	Story	Statistics of *D*_1_						Statistics of *V*_2_					
			E-W direction			N-S direction			E-W direction			N-S direction		
			*μ*	σ	*δ*	*μ*	σ	*δ*	*μ*	σ	*δ*	*μ*	σ	*δ*
1	Elastic	1	1.08	0.06	0.05	1.21	0.11	0.09	1.06	0.04	0.04	1.25	0.13	0.10
		2	1.02	0.05	0.05	1.10	0.04	0.04	1.05	0.04	0.04	1.10	0.06	0.05
		3	0.98	0.09	0.09	1.09	0.16	0.15	1.02	0.06	0.06	1.09	0.22	0.20
	Inelastic	1	1.03	0.08	0.08	1.26	0.13	0.10	1.17	0.08	0.07	1.26	0.13	0.10
		2	1.06	0.07	0.07	1.14	0.06	0.05	1.13	0.06	0.05	1.12	0.05	0.04
		3	1.03	0.11	0.11	1.10	0.18	0.16	1.06	0.08	0.07	1.12	0.19	0.17

2	Elastic	2	1.23	0.12	0.10	1.12	0.07	0.07	1.34	0.13	0.10	1.19	0.07	0.06
		3	1.13	0.09	0.08	1.04	0.07	0.07	1.04	0.08	0.08	0.99	0.06	0.06
		4	1.04	0.09	0.09	0.98	0.07	0.07	1.03	0.09	0.09	1.00	0.06	0.06
		5	1.05	0.09	0.09	1.01	0.09	0.09	1.03	0.09	0.09	1.02	0.07	0.07
		6	1.04	0.08	0.08	1.01	0.07	0.07	1.06	0.08	0.08	1.03	0.06	0.06
		7	1.05	0.09	0.08	1.01	0.08	0.08	1.02	0.08	0.08	1.00	0.07	0.07
		8	1.05	0.10	0.09	1.00	0.07	0.07	1.05	0.10	0.09	1.02	0.07	0.07
		9	1.07	0.11	0.10	1.01	0.08	0.07	1.09	0.10	0.09	1.03	0.08	0.08
		10	1.10	0.10	0.09	1.05	0.07	0.07	1.03	0.09	0.08	1.03	0.08	0.08
	Inelastic	2	1.22	0.11	0.09	1.15	0.08	0.07	1.35	0.12	0.09	1.19	0.06	0.05
		3	1.16	0.12	0.10	1.09	0.08	0.07	1.02	0.06	0.06	1.00	0.05	0.05
		4	1.06	0.12	0.11	1.01	0.08	0.08	1.03	0.07	0.06	1.02	0.06	0.06
		5	1.04	0.12	0.11	1.00	0.08	0.08	1.02	0.07	0.07	1.02	0.05	0.04
		6	1.02	0.11	0.11	0.99	0.07	0.07	1.06	0.07	0.07	1.03	0.04	0.04
		7	1.03	0.09	0.08	1.00	0.09	0.09	1.01	0.07	0.07	1.00	0.05	0.05
		8	1.03	0.10	0.09	1.03	0.09	0.09	1.06	0.09	0.08	1.04	0.06	0.06
		9	1.06	0.11	0.10	1.02	0.06	0.06	1.11	0.10	0.09	1.06	0.06	0.06
		10	1.10	0.11	0.10	1.21	0.11	0.09	1.02	0.02	0.02	1.02	0.06	0.06
